# Actuator Behaviour of Tailored Poly(thiourethane) Shape Memory Thermosets

**DOI:** 10.3390/polym13101571

**Published:** 2021-05-13

**Authors:** Francesco Gamardella, Angels Serra, Xavier Ramis, Silvia De la Flor

**Affiliations:** 1 Department of Analytical and Organic Chemistry, Universitat Rovira i Virgili, C/Marcel·lí Domingo 1, Building, N4, 43007 Tarragona, Spain; francesco.gamardella@urv.cat (F.G.); angels.serra@urv.cat (A.S.); 2 Thermodynamics Laboratory, ETSEIB Universitat Politècnica de Catalunya, Av. Diagonal, 08028 Barcelona, Spain; xavier.ramis@upc.edu; 3 Department of Mechanical Engineering, Universitat Rovira i Virgili, Av. Països Catalans, 26, 43007 Tarragona, Spain

**Keywords:** poly(thiourethane), shape-memory, thermosets, latency, actuator, single cantilever

## Abstract

In this work, a new family of poly(thiourethane) shape memory thermosetting actuators was developed and characterized. These materials can be easily prepared from mixtures of two different aliphatic diisocyanates and a trithiol in the presence of a latent catalyst, allowing an easy manipulation of the formulation. Rheological studies of the curing process confirm the latent character of the formulations. The glass transition temperatures and the mechanical properties can be modified by varying the proportion of diisocyanates (hexamethylene diisocyanate, HDI, and isophorone diisocyanate, IPDI) with stoichiometric amounts of trimethylolpropane tris(3-mercaptopropionate). The shape-memory behavior was deeply investigated under three different conditions: unconstrained, partially constrained, and fully constrained. Tests were performed in single cantilever bending mode to simulate conditions closer to real complex mechanics of thermomechanical actuators under flexural performances. The complex recovery process in single cantilever bending mode was compared with that obtained using tensile mode. The results evidenced that the amount of recovery force in fully constrained conditions, or energy released during the recovery process in partially constrained, can be modulated by simply changing the proportion of both diisocyanates. A simple model based on Timoshenko beam theory was used for the prediction of the amount of work performed. The reported results are an important guideline to design shape-memory materials based on poly(thiourethane) networks, establishing criteria for the choice of the material depending on the expected application.

## 1. Introduction

Shape-memory polymers (SMPs) are a promising class of smart materials that are capable of adopting a temporary shape and recovering their original shape upon the application of an external stimulus [1,2]. This effect usually consists in a thermomechanical process, during which the material is heated up to a temperature close to or higher than a structural transition temperature of the network (*T_trans_*) and it is deformed to this temporary shape by applying an external stress. The *T_trans_* of a SMP usually coincides with the polymer’s glass transition temperature (*T_g_*) or melting temperature (*T_m_*). *T_trans_* is an important parameter in SMP design, and it can be modified by changing the network structure of the polymer. This temporary shape can be fixed by cooling down the polymer below its *T_tran__s_*. Thus, the work carried out on the sample to change the macromolecular conformation is accompanied by a change in entropy, leaving the material in a high-energy state. After that, the original shape can be recovered by subsequently heating above *T_trans_*, and the strain energy stored during the process can be released [3,4,5].

Consequently, SMPs are capable of displacing loads during the recovery process, releasing their accumulated energy, and behaving like a smart actuator [6,7,8]. These outstanding properties have attracted great interest in recent decades, and their uses in a wide range of applications such as biomedicine [9,10], aerospace [11], textile engineering [12], and many others [13] have been studied.

Among SMPs, thermoset-based SMPs are increasingly studied for their easy processing, durability, and superior thermal and mechanical performance in comparison to common shape-memory elastomers or thermoplastic-based SMPs [14,15]. In particular, high-performance shape memory thermosets (HPSMTs) are particularly interesting for industrial applications, since they can perform mechanical work against external loads [16,17]. Our research group has contributed significantly to this field, studying and characterizing several HPSMTs based on epoxy resins [18,19,20,21].

Polyurethanes (PUs) are one of the most widely studied class of shape memory polymers, thanks to their structural versatility, easy processing, low cost, and high number of monomers available on the market [22,23,24]. Poly(thiourethane)s (PTUs) are related to their oxygen counterparts, showing comparative properties due to the presence of similar hydrogen bonding, but they did not receive the same interests from the scientific community. The formation of PTUs by nucleophilic addition of thiol to an isocyanate in the presence of a base catalyst leads to a very fast and efficient click reaction with high conversion and without the presence of unexpected groups [25]. Moreover, a high refractive index and excellent mechanical properties make poly(thiourethane)s interesting candidates for many advanced applications as optical devices, lenses, advanced coatings, and medical technology [26,27,28,29]. Despite these excellent features, the possibility of using PTUs as shape memory polymers is relatively unexplored. Only a few articles on comprehensive studies of the shape memory of PTUs have been published in recent decades [30,31,32], and their use as thermomechanical actuators is still unexplored. Our research group has already tested qualitatively the potentiality of these materials as shape memory polymers, preparing thermosets with different advanced characteristics. We prepared a family of thiol-isocyanate-epoxy materials obtained via sequential dual-curing methodology [33] and PTU covalent adaptable networks that present shape-memory characteristics combined with the possibility to modify their permanent shape [34].

Bowman and coworkers developed SMPs using the thiol-isocyanate reaction in conjunction with methacrylate homopolymerization [30] and thiol-Michael addition to form networks via two-stage polymerization [31]. Nguyen et al. synthetized urethane-thiourethane networks with shape memory properties and self-healing ability via Diels-Alder chemistry [32]. Despite these excellent papers on the subject, all these systems involved complex chemistry and multiple preparation steps, limiting their possible large-scale applications at an industrial level. Herein, we report a new type of shape memory poly(thiourethane) networks, obtained from commercially available monomers making their preparation feasible on an industrial scale.

We have synthetized a family of novel thermosets based on thiol-isocyanate “click” chemistry, in presence of a Lewis base, evaluating their performance as thermomechanical actuators. A thermal latent base (1-methylimidazolium tetraphenylborate) which releases 1MI at temperatures higher than 100 °C, previously developed by our group, has been selected as the catalyst [35,36]. The use of such latent base represents an interesting opportunity to reach temporal control of the reaction, offering the possibility to prepare the PTU thermosets in a controlled way; since the use of bases such as amines leads to a too quick thiol-isocyanate reaction, as already demonstrated in our previous paper [35]. To study the change of the material properties according to the structural characteristics of the network, PTU thermosets were prepared by varying the proportion of the flexible hexamethylene diisocyanate (HDI) and the more rigid isophorone diisocyanate (IPDI) with stoichiometric amounts of trimethylolpropane tris(3-mercaptopropionate) (S3). First, the effect of changing the diisocyanate monomer ratio on the curing schedule of the latent system was evaluated by rheological measurements. After that, a detailed thermal and mechanical characterization was performed using thermogravimetric analysis (TGA) and dynamic mechanical analysis (DMA) to have a better understanding of the relationship between network structure and thermomechanical properties. Then, to evaluate their ability to generate work during the recovery process and, as a consequence, evaluate their performance as SM actuators, the shape-memory behavior of the PTU thermosets was investigated under three different conditions: unconstrained, partially-constrained, and fully constrained. Since shape-memory actuators are commonly used as smart mechanisms for autonomous control in industrial applications, and the real operational conditions usually involve flexural instead of tension or compression actuation designs, the testing mode chosen for this study was the single cantilever mode, thus simulating an open-valve mechanism. The reported results will provide an important guideline to design shape-memory materials based on poly(thiourethane) networks, establishing criteria for choice of the material structure depending on the expected application.

## 2. Materials and Methods

### 2.1. Materials

Trimethylolpropane tris(3-mercaptopropionate) (S3), hexamethylene diisocyanate (HDI), isophorone diisocyanate (IPDI), 1-methylimidazole (1MI), and sodium tetraphenylborate (NaBPh_4_) from Sigma-Aldrich (Saint Louis, MO, USA) were used as received. The base generator, 1-methylimidazolium tetraphenylborate (BG1MI) was synthesized according to a reported methodology [36,37]. Then, 10 mmol of 1MI were solubilized in 2.6 mL of H_2_O slightly acidified with 1 mL of conc., 36% HCl solution, and 11 mmol of NaBPh_4_ were solubilized in water and stirred until complete homogenization. The two aqueous solutions were mixed, and a white salt was formed as a precipitate. The salt was filtered, washed thoroughly with distilled water and MeOH, then recrystallized from a 4:1 mixture of MeOH and CHCl_3_, filtered, and dried. The purity of the synthesized compound was assessed via differential scanning calorimetry (DSC) thermal scan and its melting point was found to be similar to the reported by other equivalent salts in the literature [38,39].

**Scheme 1:**
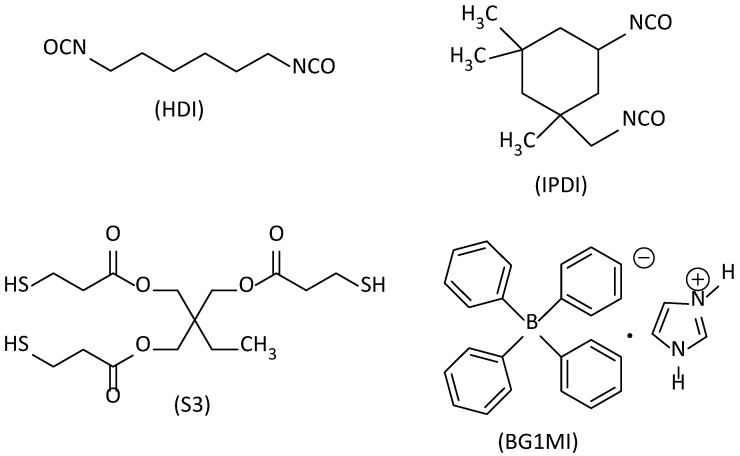
Schematic representation of the chemical substances used.

The chemical structures of the reactants used in the preparation of the materials are shown in Scheme 1.

### 2.2. Sample Reparation

The composition of the formulations with different proportions of (HDI/IPDI) with stoichiometric amount of S3 are detailed in Table 1; 0.1% wt of latent catalyst BG1MI was added to the corresponding amount of S3. The catalyst was dissolved in S3 at 80 °C until a homogeneous mixture was obtained. The mixture was poured onto aluminium moulds and cured for 2 h at 100 °C and 1 h at 125 °C with a post-curing of 2 h at 150 °C.

### 2.3. Rheological Characterization

The evolution of the curing process was monitored through rheometric measurements using a TA Instruments AR G2 (New Castle, DE, USA) rheometer equipped with electrical heated plates (EHP) in a parallel plate geometry (25 mm diameter disposable aluminium plates). To confirm the latent character of the formulations and determine the effect of the different proportions of both isocyanates on the curing, dynamo-mechanical experiments were performed at 100 °C to determine the time needed for the different formulations to reach the gel point. The rheometer oven was preheated at 100 °C and, once the temperature was equilibrated, the formulation was quickly placed between the parallel plates and the distance between the plates was settled at 1 mm. Complex viscosity (*η*) and viscoelastic properties were recorded as a function of time at three different frequencies: 1, 3, and 10 Hz. The experiments were conducted in the range of linear viscoelasticity obtained from constant shear elastic modulus (*G’*) in a strain sweep experiment at 1 Hz. Gel points were determined as the point where the viscosity diverges towards infinite.

### 2.4. Thermal Characterization

The thermal stability of the samples was evaluated by thermogravimetric analysis (TGA), using a Mettler Toledo TGA2 thermobalance (Columbus, OH, USA). Pieces of cured samples of 10–15 mg were degraded between 30 and 600 °C at 10 °C/min under inert atmosphere (N_2_ at 50 mL/min).

### 2.5. Thermo-Mechanical Characterization

A DMA Q800 (TA Instruments New Castle, USA) equipped with a 3-point-bending clamp (with a span of 15 mm) was used for the analysis of the thermo-mechanical properties of the materials. The experiments were performed at a heating rate of 3 °C/min, from 30 to 160 °C, at 1 Hz and 0.1% of strain. Prismatic rectangular samples were thoroughly polished until uniform dimensions of about 30 × 5 × 1.5 mm^3^ were obtained. The glass transition temperature *T_g_* was determined by the peak of the tan δ curve. The full width at half maximum (FWHM) was also determined as this parameter helps to quantify the homogeneity of the network structure. The values of storage modulus, *E’,* below and above glass transition were evaluated. The *T_g_^E′^* was calculated as the peak of the loss modulus. A peak in the deformability is obtained at this temperature, coinciding with the onset of the mechanical relaxation and this temperature, as explained later, is relevant in shape memory characterization.

### 2.6. Mechanical Characterization

The flexural modulus (*E*) of the materials was determined at *T_g_^E^^′^* in three-point bending mode on rectangular samples (30 × 5 × 1.5 mm^3^) using the DMA Q800. A force ramp with a constant rate of 1 N/min was imposed ensuring that only the viscoelastic behaviour of the material was evaluated. Three samples of each material were analysed, and the results were averaged.

### 2.7. Shape Memory Characterization

**Figure 1:**
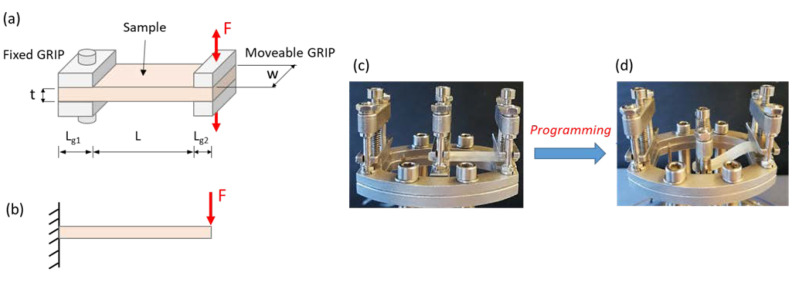
Single cantilever DMA operational mode (**a**) schematic representation and basic parameters; (**b**) mechanical model. Arrangement of the sample with its original (**c**) and programmed (**d**) shape.

To characterize the shape-memory behaviour of the PTU thermosets as bending-type thermo-mechanical actuators, the DMA Q800 single cantilever clamp was selected. DMA Q800 has the advantage in terms of accuracy of the applied load and displacement measurements in comparison with standard testing machines, when specimens with small cross sections are tested. Single cantilever mode consists of anchoring the sample on one end by the stationary clamp and attaching to a moveable clamp on the other. Through this moveable clamp, the apparatus applies a controlled force while the displacement is simultaneously recorded. It is important to emphasize that the span (L = 17.5 mm) between these two anchoring points may vary, since the moveable clamp can present a minor lateral movement and so, it should be measured prior to each test. Moreover, the clamping ends introduce shear deformation in the sample that should be taken into account when computing the relation between force and displacement. The basic characteristics of this mode as well as the equivalent mechanical model (cantilever beam with end force) are represented in Figure 1a,b. In Figure 1c,d some pictures of the real disposition of the sample (undeformed and deformed) in the clamp are also presented.

In this bending mode, the relation between the force (*F*) applied and the displacement measured in the moveable clamp (*d*) can be modelled as a first approximation using Timoshenko beam theory and correcting it by including a factor for 3D clamping effects. Consider a static linear elastic beam clamped at one end and with a point force applied at the other end where the cross-section remains parallel to the force (this last condition represents the movable clamp where the force is measured). Although in Figure 1d it can be appreciated that this is not a typical cantilever beam (where one of the ends is free), as a first approximation it can be referred as such. Using Timoshenko beam theory [40], the vertical displacement can be determined using the zero-angle boundary conditions at both ends and a zero-displacement in the clamped end. The resulting maximum displacement (*d*) takes the value:

(1) $$d=\frac{F\cdot L^{3}}{12\cdot E\cdot I\cdot \alpha_{c}}\left(1+\frac{2}{\kappa }\left(1+\upsilon \right){\left(\frac{t}{L}\right)}^{2}\right)$$

(2) $$\alpha_{c}=0.7616-0.02713\cdot \sqrt{\frac{L}{t}}+0.1083\cdot \ln\left(\frac{L}{t}\right)$$

where *d* is the maximum vertical displacement of the beam (in the tip), *F* is the force applied, and *L* is the free length between the clamped end and where the force is applied (i.e., the distance between the clamps themselves, not the distance between the midpoints of the clamps). *E* is the flexural modulus of the linear elastic material; *υ* is its Poisson’s ratio; *t* is the thickness of the cross-sectional area; *I = w·t^3^/12* is the second moment of area of the rectangular cross-section and *κ* is the Timoshenko’s shear coefficient (5/6 for a rectangular cross-section). The clamping correcting factor introduced by DMAQ800 to consider the deformation of the sample within the clamped region is *α_c_* and is given by the manufacturer.

**Figure 2:**
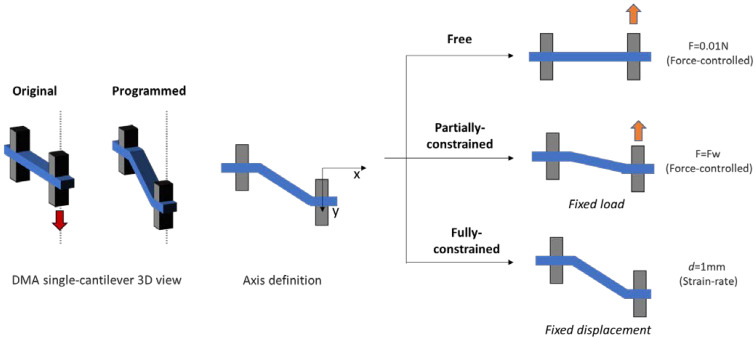
Illustration of the DMA testing depending on the experiment conditions.

Prismatic rectangular samples of 5 mm in width (*w*), 1.5 mm in thickness (*t*), and 20 mm in length have been tested in three different conditions: unconstrained, partially-constrained and fully-constrained. In all cases, there is a programming of the sample prior to the recovery process. Depending on the experiment performed in the recovery process, different DMA Q800 customized operation methods were used. The sample mounting and the recovery analysis are depicted in Figure 2. 

The temporary shape was fixed using a force-controlled mode, following the thermomechanical procedure called programming—this procedure consists of different steps. First, the specimen was heated up to the programming temperature (*T_prog_*) and deformed until reaching an imposed displacement *d_max_*. The *T_prog_* chosen was *T_g_^E^^’^*, as it is demonstrated that it is the best programming condition to store the maximum amount of entropic energy [8,17]. When *d_max_* was reached, the sample was rapidly cooled down to room temperature (well below its *T_g_*) to fix the temporary shape. After that, depending on the shape memory test, the experiments were carried out under different conditions.

For comparison purposes, the same *d_max_* must be imposed at *T_g_^E’^* to all the samples and this value must be compatible with the physical limits of the DMA. Thus, *d_max_* was chosen to be equal to 14 mm. The theoretical force F, able to produce this *d_max_*, was also calculated using Equation (1) with the flexural modulus measured at *T_g_^E’^* and compared to the experimental force needed for this displacement in order to validate the assumed Timoshenko beam theory.

For the unconstrained procedure, after the programming step, the DMA was set in force-controlled mode, the applied force was released and only a minimal force of 0.01 N was imposed to register the recovery process. The sample was heated at 3 °C/min to a temperature above *T_g_* while recording the displacement of the movable clamp *d_y_(T)*, thus measuring the shape-memory recovery process (SR) determined by the following equation:

(3) $$\mathrm{SR}(T)=\frac{d_{max}-d_{y}\left(T\right)}{d_{max}}$$

where *d_max_* is the programmed displacement and *d_y_(T)* is the displacement reached by the movable clamp during the recovering process. The recovery process ended with a displacement *d_y_(T_end_)* which represents the amount of deformation that the sample is not able to recover. These curves were normalized for comparison purposes (0 means no recovery and 1 means completely recovered).

To analyse the effect of the different formulations on the shape-recovery process the shape-recovery curves *SR(T)* were differentiated with respect to the temperature by using equation (4) thus obtaining the instantaneous shape-recovery speed *SR_speed_(T)* as a measure of the shape-recovery sharpness. Using the *SR_speed_(T)* curve, the temperature corresponding to the maximum recovery speed was determined as the peak of the curve (*T_peak_*).

(4) $$SR_{speed}(T)=\frac{dSR\left(T\right)}{dT}$$

The shape-recovery rate (*V_r_*), a measure of the average shape-recovery speed, was evaluated from *SR(T_1_)* = 0.15 to *SR(T_2_)* = 0.85, that is in the range between 15% and 85% of the shape recovery process (i.e., avoiding the early and final stages) using Equation (5):

(5) $$V_{r}\left(\%/\min\right)=\frac{SR_{15\%-85\%}}{\Delta t_{SR_{15\%-85\%}}}$$

Finally, the recovery-ratio was calculated by Equation (6):

(6) $$R_{r}=\left(1-\frac{d_{y}\left(T_{end}\right)}{d_{max}}\right)\cdot 100$$

Fully-constrained experiments were performed to calculate the maximum recovery force that the PTUs were able to generate. To do that, the experiments were carried out in strain-rate mode imposing a minimum constant displacement (1.0 μm) while heating the sample at 3 °C/min. The force generated by the sample, *F_y_(T),* was measured during the recovery process, until reaching a maximum value *F_max_.*

To evaluate the mechanical work generated, partially-constrained experiments were performed in force-controlled mode applying a constant force (*F_w_*) equal to the 50% of the force generated for each sample during the fully-constrained experiments (*F_max_)*. Then, the sample was heated up to the recovery temperature while the displacement of the movable clamp was measured. The relative work (*W_rel_*) developed by the sample was calculated using Equation (7). In this expression, for comparison purposes, the final displacement reached, *d_y_(T_end_)* has been normalized with respect to the maximum displacement, *d_max_* imposed during programming.

(7) $$W_{\mathrm{rel}}=F_{w}\cdot \frac{d_{max}-d_{y}\left(T_{\mathrm{end}}\right)}{d_{max}}$$

In Equation (7) *F_w_* is the constant force applied on the sample, *d_max_* is the deflection after the programming process, and *d_y_* (*T_end_*) is the displacement reached by the movable clamp at the end of the recovery process.

The theoretical relative work that the sample could perform can be deduced assuming that all the force produced by the sample during the shape-recovery process is equal to the force given to program the sample in the temporary-shape (*F_prog_*). *F_prog_* can be deduced, as explained before, from Equation (1) using Timoshenko beam theory assuming *d_max_* = 14 mm (the maximum deflection imposed in programing) and with the flexural modulus measured at *T_g_^E’^*. Once *F_prog_* is determined, the theoretical displacement that the sample could reach in the recovery process, *d_y-theor_* (*T_end_*), can be determined again by Equation (1) but considering that the force applied by the DMA, *F_w_*, acts in opposite direction during the recovery experiment. Thus, in equation (1), *F* is calculated as *F = F_prog_-F_w_*. Finally, the theoretical relative work can be calculated as:

(8) $$W_{\mathrm{rel}\text{-}\mathrm{theor}}=F_{w}\cdot \frac{d_{max}-d_{y\text{-}theor}\left(T_{\mathrm{end}}\right)}{d_{max}}$$

For a better comprehension of the stress-strain behaviour in single cantilever bending, the shape-memory behaviour under unconstrained conditions was also analysed using a tension-film clamp (the simplest stress-state possible in mechanics) and compared with the behaviour in bending mode. The experiments were conducted in controlled-force mode on rectangular specimens with dimensions of 20 × 5 × 0.5 mm^3^. Under this tensile mode, the sample was heated up to the programming temperature and loaded at 1 N/min until a programming stress (*σ_prog_*) equal to 75% of the stress at break was reached (*σ_prog_* = 0.75·*σ_break_*). The permanent shape was recovered by heating the programmed sample at 3 °C/min to a temperature above *T_g_*. The recovery process ended with a deformation value of *ε_p_*, which represents the amount of deformation that the sample was not able to recover. The shape-memory recovery (SR) was determined using Equation (9).

(9) $$\mathrm{SR}(T)=\frac{\varepsilon_{max-}\varepsilon_{p}}{\varepsilon_{max}}\cdot 100$$

where *ε_max_* is the deformation corresponding at the 75% of the strain at break and *ε_p_* is the value of the deformation at the end of the recovery process.

## 3. Results

Two different isocyanates were selected as starting monomers, hexamethylene diisocyanate (HDI) with a linear and flexible structure and isophorone diisocyanate (IPDI), more rigid due to the presence of a cyclohexane ring in the structure. Both were crosslinked with a trifunctional thiol (S3) in the presence of a base to obtain PTUs with different mechanical and thermal properties. Fixing the thiol at the stoichiometric proportion and the amount of catalyst in the formulation and varying only the proportion of both aliphatic diisocyanates, the influence of the compositional ratio on the dynamic mechanical and thermal properties of poly(thiourethane) networks can be analysed.

### 3.1. Rheological Study

**Figure 3:**
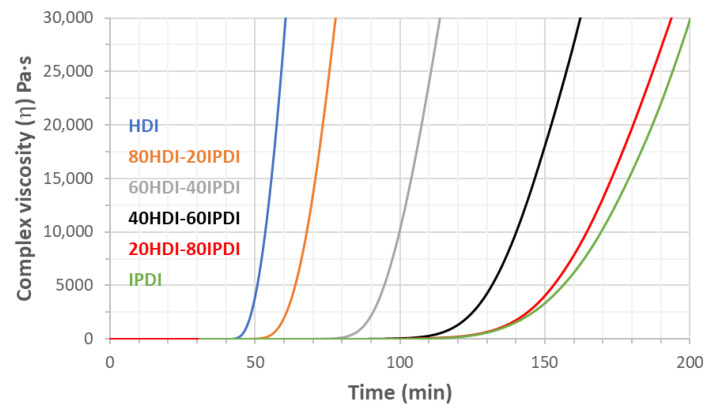
Time sweep experiment for the different formulations prepared at 100 °C.

As the first step of the material characterization, the rate of the network formation was analysed by means of a rheological study. During the curing process, the physical state of the formulation changes from a liquid to a sol/gel rubber and then to a glassy or solid and the rate at which this occurs depends on several factors such as: monomer structure, catalyst type, amount of catalyst, curing temperature, etc., all of them affecting the processing conditions [41]. The gel point represents the endpoint for a formulation to flow in a mould or to be applied in an industrial process. After the gel point, it is difficult or impossible for the mixture to be manipulated, and therefore the time at which it occurs has great technological relevance. With this rheological study, we aim to demonstrate the latent character of the formulation and evaluate the effect of changing the diisocyanate monomer ratio on the gel time of the system. The isothermal curing processes of the different formulations prepared were investigated in the rheometer, to obtain the polymerization time dependence on the diisocyanate proportion, determining the time gel at 100 °C. The changes in complex viscosity (*η*) as a function of time at 100 °C for all the formulations are shown in Figure 3.

As we can see in Figure 3, at the beginning of the curing stage the viscosity remains constant for some time, and then, at a certain point, a rapid increase in viscosity is observed, demonstrating the latent character of the formulations. The critical gel time was determined as the time in which the viscosity diverges towards infinity without reaching a steady state [42]. The gel times obtained are given in Table 2.

The gelation time depends on the stoichiometry of the formulation since the reactivity of both diisocyanates is different. The more flexible structure of HDI, with respect to IPDI, presents greater mobility which can affect the rate of the curing reaction. In the HDI both isocyanate groups are linked to a methylene carbon, but in IPDI one of the isocyanates is linked to a methine carbon in the cyclohexane ring, which can experience steric hindrance to the attack to the thiol groups, reducing in this way its reactivity. According to these factors, the time to reach the gelation increases with the proportion of IPDI in the reactive mixture.

### 3.2. Thermal Degradation Study

**Figure 4:**
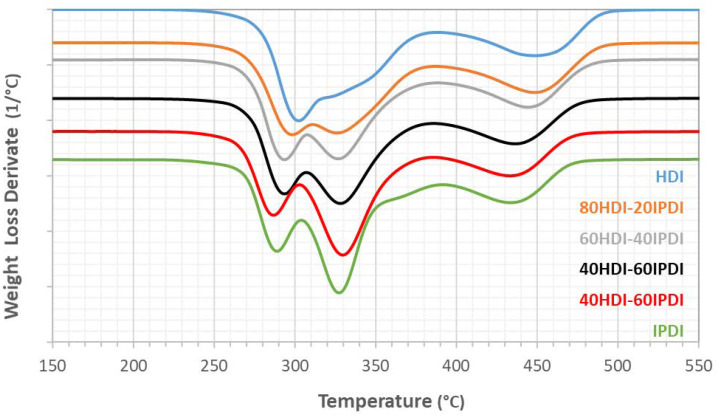
DTG curves of the poly(thiourethane)s prepared (the curves are shifted for a better understanding).

A thermal degradation study was performed to ensure that all the samples are fully stable in the temperature range used in the shape memory process. The thermal stability of the PTUs was analysed by TGA and the derivative curves of the TGA degradation are shown in Figure 4, where the overlapped curves are shifted for a better understanding. The initial degradation temperature (*T_2%_*) extracted from these experiments are collected in Table 2. All of them present a high thermal stability, with a *T_2%_* around 270 °C for all samples, with a slight decrease on increasing IPDI content in the samples. Figure 4 shows that there are barely differences in the degradation evolution for all the materials evaluated.

As we can see in Figure 4, the degradation mechanism is quite complex, presenting three different stages. The first degradation step is attributed to the carbonyl sulphide elimination, as already demonstrated by Rogulska et al. [43] and by our group, with a maximum placed around 300 °C [36]. This first peak is shifted to higher temperature when increasing the amount of HDI. The second step is associated with the decomposition of the ester bond of the thiol structural units. First and second peaks of degradation are more overlapped on increasing the proportion of HDI in the formulation. The third degradation peak corresponds to the full degradation of the network. From the degradation study, we can state that all these materials are fully stable up to temperatures around 250 °C and, consequently, in the entire working temperature range in the shape memory behaviour.

### 3.3. Thermomechanical Analysis

**Figure 5:**
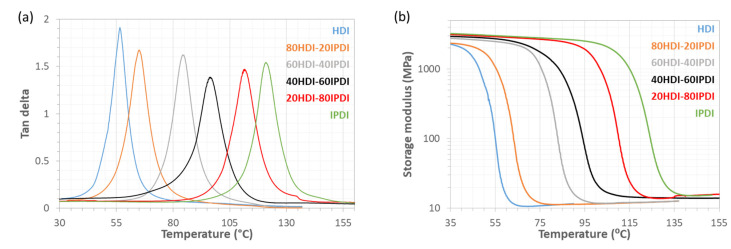
(**a**) Evolution of *tan δ* and (**b**) storage modulus with temperature of the different poly(thiourethane) samples.

To determine the effect of changing the proportion of both aliphatic diisocyanates on the network homogeneity and on the glass transition temperature, dynamic thermomechanical analyses of PTUs were performed. The resultant plots are shown in Figure 5 and the main data extracted from thermomechanical tests are summarized in Table 2.

As shown in Table 2, the glass transition temperatures and the storage moduli at glassy state can be tailored by varying the proportion of both isocyanates in the samples. As the content of IPDI increases, due to its high rigidity, higher values of *T_g_* and *E’_g_* are obtained. By mixing both diisocyanates, we obtained a wide range of glass transition temperatures between 57 °C and 125 °C, referred to as the tan delta peak. The storage modulus at 30 °C significantly increases as the IPDI content increases, from 2.3 to 3.2 GPa, moving from the more flexible structure to the rigid one.

All these thermomechanical changes were obtained without affecting the homogeneity of the sample as shown by the very narrow *tan δ* curves in Figure 5. The slight decrease of the height of *tan δ* curve for samples prepared with higher content of IPDI is indicative of a more densely crosslinked network with more restricted mobility. The high homogeneity of the samples is derived from the click character of the thiol-isocyanate reaction catalysed by a base, and it is demonstrated by the low values of the width of the curve at half height (FWHM) in the range between 8.5 °C and 13 °C. To achieve good fixation and fast recovery, a high homogeneity of the material is crucial for shape-memory applications, because the narrower the transition, the faster the recovery process.

In terms of shape-memory properties, the *E’_g_/E’_r_* ratio is higher than 100 in all cases. This means two orders of magnitude, which is high enough to expect good shape-memory properties in terms of shape-fixation and shape-recovery. This parameter increases as the proportion of IPDI increases. In all the materials the value of the rubbery modulus *E’_r_* is similar regardless of the structural differences, therefore the *E’_g_/E’_r_* ratio is mainly governed by *E’_g_*.

In light of these considerations, we can state that the main thermomechanical properties such as *T_g_*, *T_g_^E’^* or storage moduli *E’_g_* of the PTUs can be easily tuned by changing the structure and composition of the isocyanate monomer. These changes were achieved by maintaining the uniformity of the network associated with thiol-isocyanate polymeric materials. Therefore, we can obtain different materials with a wide range of thermomechanical properties that meet the requirements in terms of *E’*_g_/*E’_r_*, and that can present exciting characteristics for shape-memory applications.

### 3.4. Shape-Memory Results

#### 3.4.1. Unconstrained Recovery

**Figure 6:**
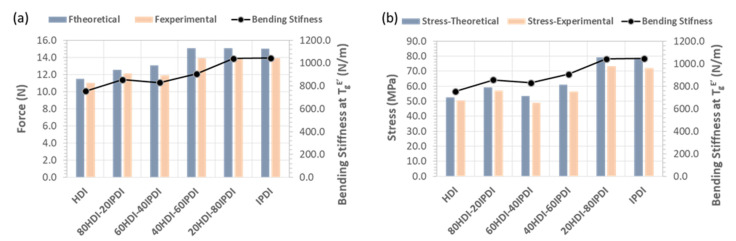
Theoretical and experimental forces (**a**) and stresses (**b**) in the programming process. Bending stiffness obtained for each sample is also indicated.

The shape-memory properties, as explained in the experimental part, were analysed in three different conditions. First of all, the unconstrained recovery experiments were carried out to evaluate the shape-memory effect of the different PTUs prepared. This shape memory characterization consists of two steps: programming of temporary shape and recovery of the original shape without any constraints. The maximum displacement, *d_max_*, imposed in programming, was set to 14 mm and, consequently, for comparison purposes, the dimensions (thickness and width) in all the samples were the same. The theoretical force *F* generated during programming was computed with Equation (1), with the bending stiffness measured at *T_g_^E’^* for each sample, and this force was compared to the experimental one. In Figure 6 this comparison, together with the bending stiffness at *T_g_^E’^*, is presented. As it can be observed in Figure 6a, in all the samples, the predicted forces are higher than the experimental ones. This is because the theoretical model predicts a linear behaviour, in which *F* and *d_max_* are proportional while, but the stress-strain behaviour at *T_g_^E’^* is viscoelastic and only linear and proportional during the initial part. Nevertheless, the assumption of the Timoshenko beam model can be used as an upper boundary limit for a simple initial prediction. It is worth noting that for the 20HDI-80IPDI and IPDI, due to the high rigidity of the samples, the predicted forces were higher than the limits of the DMA (18 N). To avoid this problem, in these two samples, the thickness was reduced to 1.3 mm instead of 1.5 mm. That is the reason why the forces in 40HDI-60IPDI, 20HDI-80IPDI, and IPDI are very similar. To avoid these small differences in dimensions, the stress corresponding to this force was calculated using bending stress-strain relations and represented in Figure 6b. This relation can be only used for comparison purposes because, as it will be demonstrated later, the stress-state along the sample is much more complex.

**Figure 7:**
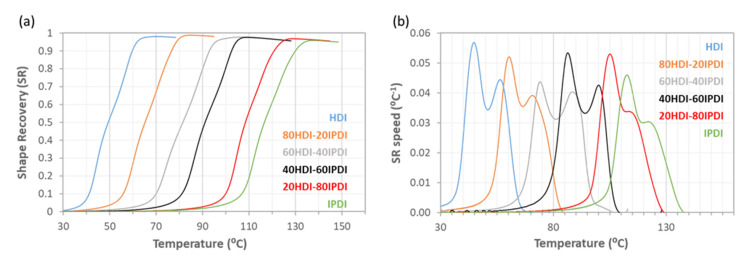
(**a**) Shape-memory unconstrained recovery experiment, shape-recovery (*SR*) against temperature. (**b**) Derivative of the shape-recovery with respect to temperature (*SR_speed_*) as function of temperature.

The free-recovery processes for all the samples are shown in Figure 7, in which the shape-recovery (*SR*) and the recovery speed (*SR_speed_*) are plotted as function of the temperature. The main parameters obtained from these tests are presented in Table 3. It is important to notice that all the samples were able to recover the original shape, with shape-recovery ratios, *R_r_*, close to 100%. The value of *R_r_* slightly decreases as the IPDI content increases, obtaining the lowest *R_r_* with the material completely based on IPDI. This is probably due to a stress hardening process taking place during the loading process that generates permanent deformation in the network structure after programming the temporary shape [15].

The shape-recovery speed curves (*SR_speed_*) presented in Figure 7b show that the materials prepared with high content of HDI are slightly faster than the materials based on IPDI. The values of recovery rate (*V_r_*), presented in Table 3, increase from 11.8%/min for IPDI to 13.5%/min in HDI sample. As expected, the increase of the content of IPDI in the formulation produces a decrease in *V_r_* due to the lower mobility of the network structure and the slightly higher heterogeneity also highlighted by the FWHM parameter.

**Figure 8:**
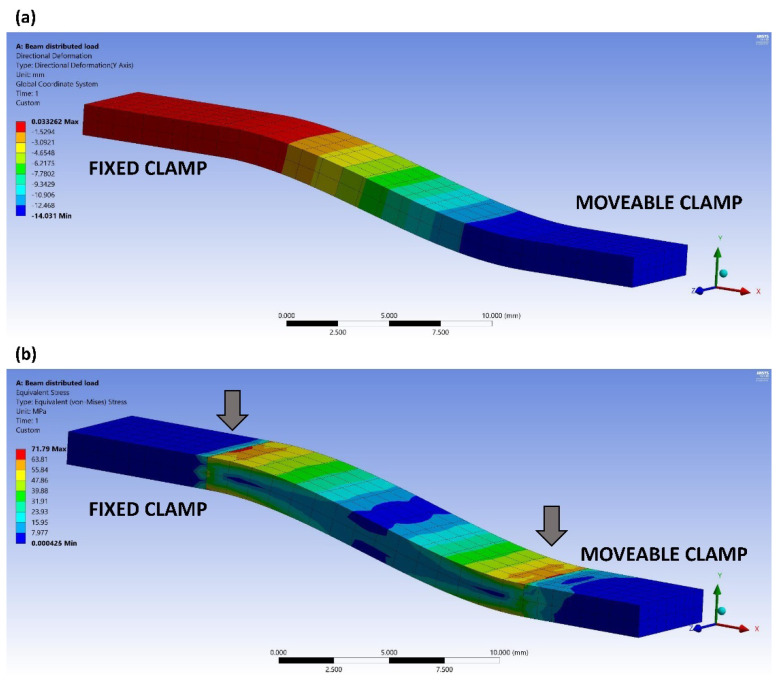
Finite Element Modelling of the programmed sample 60HDI-40IPDI (d_max_ = 14 mm) in single cantilever mode using classical beam theory. (**a**) Deformed beam after programming and (**b**) Von Mises stress distribution where the points of maximum stress are indicated with arrows.

The curves of the derivative of the shape-recovery shown in Figure 7b present a different shape with respect to the one usually observed when testing in tensile mode. These curves present two peaks (*T_I-peak_* and *T_II-peak_* in Table 3) reflecting the temperatures where the recovery process is maximum. These temperatures are strictly related to the glass transition temperature of the material and are located close to the values of *T_g_^E’^* and *T**_g-tan δ_*** (see Table 2). The fact that these curves present two maximum peaks instead of only one, like in tensile mode, is probably related to the complex stress state suffered by the samples in single cantilever bending mode. This is also evident in Figure 7a where the curves present a slight shoulder in the middle of the recovery process. During the programming process in this stress-state mode, unlike in tension mode, the stress distribution is not uniform along the beam, being that the maximum programming bending stress located near the fixed and moveable clamp while the minimum stress is located in the centre of the beam. To demonstrate this stress concentration effect, the mechanical behaviour of the sample 60HDI-40IPDI programmed at *T_g_^E’^* was modelled using the classical beam theory in Finite Element Analysis using Ansys^®^ Academic Research Mechanical, Release 2019R2. It is important to mention that the moveable clamp measures L_g2_ = 6.35 mm, while the fixed clamp measures L_g1_ = 7.625 mm and the load applied in the deformation process is distributed along L_g1_, as observed in Figure 1a. Therefore, these lengths should be taken into account when modelling the imposed displacement in the moveable clamp and the fixed support in the Finite Element Modelling simulation. The programmed sample (directional displacement of 14 mm) is shown in Figure 8a. In Figure 8b the resultant stress state due to the programming (Von Mises stress) is shown, where the maximum stress locations are pointed out. Note that the stress obtained by the simulation is slightly higher than the theoretical one, as plotted in Figure 6b, because of the differences in the way the displacement is applied, the constraints, and the second-order effects.

To demonstrate that the shape of the SR curves (Figure 7) in the free recovery process is related to the complex stress-state to which the sample is subjected in single cantilever mode and not to the network structure of the material, the shape memory effect was also evaluated in tension mode, using the film-tension clamp, and compared with the results previously obtained.

**Figure 9:**
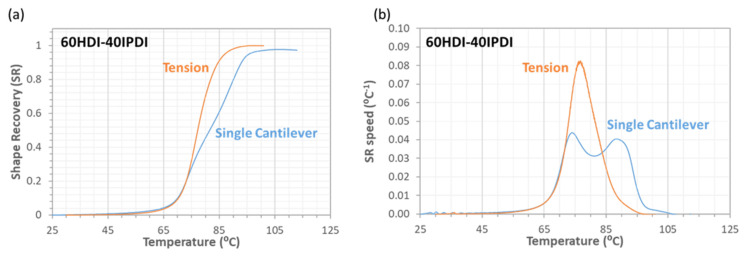
Shape-memory unconstrained recovery experiment with tensile and single cantilever clamp for the material prepared with S3–60HDI-40IPDI: (**a**) Shape Recovery vs. temperature and (**b**) (*SR_speed_*) vs. temperature.

For this comparison, we selected the material with 60% of HDI and 40% of IPDI to have at the same time the presence of both isocyanates. The comparison of the two recovery processes can be observed in Figure 9.

As can be seen in Figure 9, the recovery process in tensile mode is uniform with a progressive evolution, clearly visible in the derivative of the shape recovery, which presents only one peak at 77 °C. This SR process is faster in tensile (*V_r_* = 19.1%/min) compared to single cantilever-bending mode (*V_r_* = 11.1%/min), due to the simplicity in the stress and strain state along the sample in the first mode. Nevertheless, the mechanics of a complex SMP actuator look like a cantilever mode rather than a tensile mode (for example, in functional mechanisms like flexural jointed mechanisms and other robotic systems, especially in the micro-domain). These results put in evidence that the use of the single cantilever clamp affects the shape recovery process, producing two different recovery phenomena as evidenced from the derivative of SR, even if in both cases the materials were able to recover their original shape.

#### 3.4.2. Fully-Constrained Recovery Tests

**Figure 10:**
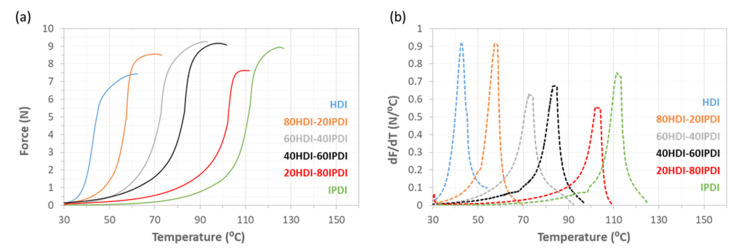
(**a**) Force generated with increasing temperature in fully-constrained recovery experiments; (**b**) derivative of the force generated with respect to the temperature (*dF/dT*), as function of temperature.

To determine the maximum forces generated by the SMPs under a completely impeded scenario, fully-constrained experiments were carried out. The curves of the force generated, and the force rate calculated against temperature, *dF/dT*, are presented in Figure 10. The parameters obtained are summarized in Table 3. 

The force generated is strictly related to the network structure, more densely crosslinked networks usually produce higher driving forces being the crosslinking density the most important factor in the generation of the recovery force. Since the crosslinking density is related to the value of the modulus in rubbery state, materials with a higher value of relaxed modulus, usually, are able to generate higher recovery forces [18], if no hardening effects are present. In our case, as the relaxed moduli (Table 2) are very similar for all the PTUs prepared, the forces generated are expected to be similar for all the materials. As can be observed in Figure 10a, during the recovery process the forces progressively increase with temperature until reaching a maximum value. These maximum forces produced are relatively high and similar for all the materials, reaching values between 7.4 N and 9.3 N. For materials with lower content of IPDI, as *E’_r_* is slightly lower, the forces generated are also slightly lower (7.4 N and 8.5 N). As the IPDI content increases, *F_max_* increases (around a maximum of 9.3 N for sample 40HDI-60IPDI). For samples with the highest content of IPDI, although with a similar *E’_r_* to 40HDI-60IPDI, *F_max_* is limited, probably due to a strain hardening process that may cause small irreversible plastic deformation in the network structure.

In a similar way to the *SR_speed_*, the force rate, *dF_y_/dT* in (N/°C), is plotted in Figure 10b. Paying attention to the peak of these curves, it is clear that the *T_peak_* is strictly related to the glass transition temperature of the materials, since the maximum rate of force generated is located close to the *T_g_^E’^*, according to the unconstrained experiments.

**Figure 11:**
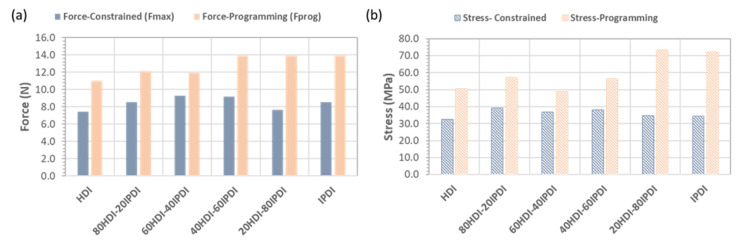
(**a**) Maximum force generated in fully-constrained recovery experiments (*F_max_*) compared to the maximum force needed in programming (*F_prog_*); (**b**) for comparison purposes these forces have been made independent from dimensional parameters thus calculating the corresponding stresses.

The force applied for programming the samples were also compared with the force generated in fully constrained conditions. In Figure 11, these values for each sample in terms of force (Figure 11a) and stress (Figure 11b) are presented. The stress generated by the PTUs were calculated using bending stress-strain relations with the purpose of eliminating the influence of sample dimensions. In terms of efficiency, all the samples except those with higher proportion of IPDI “lost” around 25–30% of the stress. Samples 20HDI-80IPDI and IPDI lost around 53% probably due to the hardening process and plastic deformation that took place during the loading process, as already mentioned. The values of stress must be considered only for comparison purposes (to obtain values independent of dimensions) because, as stated previously, the stress-state is much more complex. 

These results are considerably higher than the ones reported by Belmonte et al. [21] using thiol-epoxy shape-memory actuators under 3-point bending conditions. Although the values of *E_r_^’^* in [21] are considerably higher than PTUs values of *E_r_^’^*, the force generated by the PTUs are higher than the values reported by Belmonte et al. which were not greater than 3 N (or 6 MPa in terms of stress to obtain values independent of dimensions).

### 3.5. Partially-Constrained Recovery Tests

Partially-constrained experiments were performed to fully characterize the PTU thermosets as actuators, because the primary use of a polymer actuator is to recover its shape by working against a force, thus developing work.

**Figure 12:**
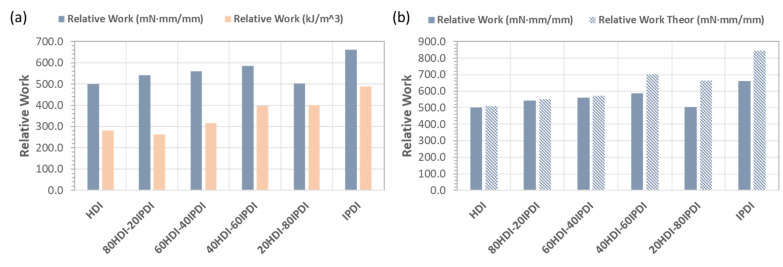
(**a**) Experimental relative work output for all the PTUs prepared; (**b**) comparison between experimental and theoretical relative work generated.

In this test, a constant force, *F_w_*, was imposed at the moveable clamp and the work generated during the recovery-process was calculated using Equation (8). To avoid the break of the sample, the force applied was calculated as the 50% of the maximum force generated during the fully-constrained experiments (i.e., *F_w_* = 0.5 × *F_max_)*. It is important to notice that all the samples were able to produce positive work in the shape recovery process, even if the force applied on the samples was greater than 3.5 N. The values obtained from the partially-constrained experiments are presented in Figure 12a. For a better comparison, the stress corresponding to *F_w_* and the strains corresponding to the displacements can be simply computed with bending stress-strain relations. Thus, the work developed during the constrained recovery can be calculated per unit volume (kJ/m^3^) as:

(10) $$W_{\mathrm{rel}}\left(kJ/m^{3}\right)=\sigma_{w}\cdot \left(\varepsilon_{dmax}-\varepsilon_{dy\left(Tend\right)}\right)$$

The amount of work generated presents comparable values for all the materials prepared. Among them, the material with 100% IPDI was able to generate the best result in terms of work, reaching a maximum value of around 660 mN*m/m (around 490 kJ/m^3^) due to its higher rigidity. The thermomechanical parameter that governs the work developed is *E’_g_/E’_r_.* Although in the IPDI sample, the applied *F_w_* is comparatively lower with respect to other PTU samples (due to its relative lower *F_max_*), the work generated is the highest due to the highest *E’_g_/E’_r_* ratio. The values obtained with the poly(thiourethane) thermosets are considerably higher than the values reported by Belmonte et al. [21] with dual cured thiol-epoxy polymers, who obtained a maximum value of 314 mN*mm/mm in 3-point bending conditions.

As explained in the experimental part, the maximum theoretical work that the materials are able to generate can be estimated assuming that the sample produces a force during the recovery process equal to *F_prog_*, i.e., there are no energy losses during programming and therefore all programmed force is released later during the recovery-process. In Figure 12b the comparison between the experimental and theoretical work is presented. It can be observed that the Timoshenko beam theory predicts an upper maximum limit that fits quite well with the work developed by the samples, given the best adjustment for samples with lower proportion of IPDI. As explained in free-recovery experiments, the predicted *F_prog_* is higher than the experimental ones because the model assumes a linear and proportional behaviour, without any strain hardening effect that probably takes place in samples with higher proportion of IPDI and that reduces the recovery process.

These results indicate that all the poly(thiourethane) thermosets synthetized exhibit good performance during the partially-constrained experiments in single cantilever mode. In this way, the possibility of obtaining actuators with different activation temperatures by varying the *T_g_* has been demonstrated by only changing the diisocyanate structure, so that they better match with potential future applications without influencing their ability to perform a work.

## 4. Conclusions

In this work we developed a new family of shape-memory actuators based on poly(thiourethane) networks using two different aliphatic isocyanates, isophorone diisocyanate (IPDI) and hexamethylene diisocyanate (HDI), and a trifunctional thiol (S3) acting as the crosslinker. The curing of thiol-isocyanate formulations was influenced by the proportion and structure of the diisocyanate monomers. The use of 1-methylimidazolium tetraphenylborate (BG1MI), as a latent base catalyst, allowed a control of the reaction using both aliphatic diisocyanates. The materials showed high homogeneity within a broad range of glass transition temperatures between 50 and 120 °C. The *T_g_* values increased upon increasing the content of the rigid IPDI monomer.

The SMP behaviour of the poly(thiourethane)s were fully characterized as potential actuators in single cantilever bending mode to reproduce real mechanics of complex SMP actuators as in functional mechanisms. Three different scenarios were tested: unconstrained, partially constrained, and fully constrained. Free-recovery experiments showed an efficient recovery process for all the materials prepared. Fully-constrained recovery experiments indicated a notable capacity of these materials to generate force, with a maximum value obtained higher than 9 N, thanks to the combination of the two different diisocyanate monomers selected. Partially-constrained recovery experiments demonstrated the ability of these materials to produce high levels of work output, despite the complex stress and strain state caused by the single cantilever clamp. Using classical Timoshenko beam theory, the maximum potentiality of these materials can be efficiently predicted as a first approximation both in programming, unconstrained and partially constrained conditions.

In conclusion, we have demonstrated the ability of this new series of poly(thiourethanes) to be used as smart materials and the possibility to combine different diisocyanate monomers to tune the thermomechanical properties without affecting the homogeneity of the sample and preserving the shape-memory ability.

## Figures and Tables

**Table 1:** Composition of the formulations prepared by changing the HDI/IPDI proportion in molar and weight percentages. The thiol (S3) is added in stoichiometric proportions and the latent base in 0.1 phr in reference to the thiol amount.

Sample	Mole Ratio (HDI/IPDI) (%)	Weight Ratio (S3/HDI/IPDI) (%)
HDI	100:0	61:39:0
80HDI-20IPDI	80:20	60:30:10
60HDI-40IPDI	60:40	58:22:20
40HDI-60IPDI	40:60	57:14:29
20HDI-80IPDI	20:80	56:7:37
IPDI	0:100	54:0:46

**Table 2:** Rheological, thermal (TGA) and thermomechanical (DMA) data obtained for the PTU samples. Coefficients of variation less than 3% for TGA results and temperatures in DMA data and less than 5% for all the moduli.

	Rheometer	TGA	DMA					
Sample	Gel Time ^1^ (min)	T_2%_ ^2^ (°C)	*T_g_^E’^* ^3^ (°C)	*T_g-tanδ_* ^4^ (°C)	*E’*_g_ ^5^ (GPa)	*E’_r_* ^6^ (MPa)	FWHM ^7^ (°C)	*E’*_g/_ *E’_r_*
HDI	48	276	44	57	2.3	10	8.5	164
80HDI-20IPDI	58	273	55	65	2.3	12	10	176
60HDI-40IPDI	83	269	75	85	2.8	12	11	200
40HDI-60IPDI	120	269	84	97	2.9	13	13	223
20HDI-80IPDI	133	269	97	108	3.0	14	13	214
IPDI	137	267	114	125	3.2	13	11	246

^1^ Gel point determined at the time when the viscosity diverges toward infinity; ^2^ Temperature of 2% of weight loss determined by TGA in N_2_ at 10 °C/min; ^3^ Temperature of the peak of the loss modulus; ^4^ Temperature of maximum of the tan δ; ^5^ Glassy modulus determined at 25 °C; ^6^ Relaxed modulus determined at the T_tan δ_ + 30 °C (in the rubber state); ^7^ Full width at half maximum of tan δ.

**Table 3:** Parameters obtained from the shape-memory tests (unconstrained, fully-constrained and partially-constrained experiments) of the different materials: Recovery-rate (*R_r_*), shape-recovery speed (*V_r_*), peak temperature of the derivate of the shape recovery (*T_peak_*)_,_ shape-recovery force (F_max_), relative work (W_rel_). Coefficients of variation less than 5%.

	Unconstrained				Fully-Constrained		Partially- Constrained
Sample	*R_r_* (%)	*V_r_* (%/min)	*T_I-peak_* (°C)	*T_II-peak_* (°C)	*F_max_* (N)	*T_peak_* (°C)	*W_rel_* mN/(mm/mm)
HDI	98.0	13.5	44.8	57.0	7.4	42.8	501.5
80HDI-20IPDI	98.0	12.4	60.3	70.8	8.5	58.6	542.7
60HDI-40IPDI	97.5	11.1	74.0	88.2	9.3	72.4	560.5
40HDI-60IPDI	97.6	12.3	86.3	99.9	9.1	84.8	585.9
20HDI-80IPDI	96.8	11.9	105.0	111.2	7.6	102.0	503.1
IPDI	95.9	11.8	112.6	122.0	8.5	111.8	660.9

## Data Availability

Not applicable.

## References

[1] Hager M.D., Bode S., Weber C., Schubert U.S. (2015). Shape memory polymers: Past, present and future developments. Prog. Polym. Sci..

[2] Zhao Q., Qi H.J., Xie T. (2015). Recent progress in shape memory polymer: New behavior, enabling materials, and mechanistic understanding. Prog. Polym. Sci..

[3] Hu J., Zhu Y., Huang H., Lu J. (2012). Recent advances in shape–memory polymers: Structure, mechanism, functionality, modelling and applications. Prog. Polym. Sci..

[4] Souri M., Lu Y., Erol A., Pulla S., Karaca H. (2015). Characterization of unconstraint and constraint shape recoveries of an epoxy based shape memory polymer. Polym. Test..

[5] Lendlein A., Gould O.E.C. (2019). Reprogrammable recovery and actuation behaviour of shape-memory polymers. Nat. Rev. Mater..

[6] Berg G.J., McBride M.K., Wang C., Bowman C.N. (2014). New directions in the chemistry of shape memory polymers. Polymer.

[7] Anthamatten M., Roddecha S., Li J. (2013). Energy Storage Capacity of Shape-Memory Polymers. Macromolecules.

[8] Belmonte A., Guzmán D., Fernández-Francos X., De la Flor S. (2015). Effect of the network structure and programming temperature on the shape-memory response of thiol-epoxy “click” systems. Polymers.

[9] Liu R., Xu S., Luo X., Liu Z. (2020). Theoretical and Numerical Analysis of Mechanical Behaviors of a Metamaterial-Based Shape Memory Polymer Stent. Polymers.

[10] Hardy J.G., Palma M., Wind S.J., Biggs M.J. (2016). Responsive Biomaterials: Advances in Materials Based on Shape-Memory Polymers. Adv. Mater..

[11] Liu Y., Du H., Liu L., Leng J. (2014). Shape memory polymers and their composites in aerospace applications: A review. Smart Mater. Struct..

[12] Hu J., Chen S. (2010). A review of actively moving polymers in textile applications. J. Mater. Chem..

[13] Pilate F., Toncheva A., Dubois P., Raquez J.-M. (2016). Shape-memory polymers for multiple applications in the materials world. Eur. Polym. J..

[14] Xie F., Huang L., Leng J., Liu Y. (2016). Thermoset shape memory polymers and their composites. J. Intell. Mater. Syst. Struct..

[15] Belmonte A., Fernández-Francos X., De la Flor S. (2017). New understanding of the shape-memory response in thiol-epoxy click systems: Towards controlling the recovery process. J. Mater. Sci..

[16] Karger-Kocsis J., Kéki S. (2017). Review of Progress in Shape Memory Epoxies and Their Composites. Polymers.

[17] Russo C., Fernández Francos X., De la Flor S. (2020). Shape-memory actuators based on dual-curing thiol-acrylate-epoxy thermosets. Express Polym. Lett..

[18] Belmonte A., Fernández-Francos X., De la Flor S. (2018). Thermomechanical characterization of thiol-epoxy shape memory thermosets for mechanical actuators design. AIP Conf. Proc..

[19] Santiago D., Fabregat-Sanjuan A., Ferrando F., De La Flor S. (2016). Recovery stress and work output in hyperbranched poly(ethyleneimine)-modified shape-memory epoxy polymers. J. Polym. Sci. Part B Polym. Phys..

[20] Belmonte A., Lama G.C., Gentile G., Cerruti P., Ambrogi V., Fernández-Francos X., De La Flor S. (2017). Thermally-triggered free-standing shape-memory actuators. Eur. Polym. J..

[21] Belmonte A., Russo C., Ambrogi V., Fernández-Francos X., De la Flor S. (2017). Epoxy-based shape-memory actuators obtained via dual-curing of off-stoichiometric “thiol-epoxy” mixtures. Polymers.

[22] Jahid M.A., Hu J., Wong K., Wu Y., Zhu Y., Sheng Luo H.H., Zhongmin D. (2018). Fabric Coated with Shape Memory Polyurethane and Its Properties. Polymers.

[23] Kausar A. (2017). Review on Technological Significance of Photoactive, Electroactive, pH-sensitive, Water-active, and Thermoresponsive Polyurethane Materials. Polym. Plast. Technol. Eng..

[24] Song X., Chi H., Li Z., Li T., Wang F. (2020). Star-Shaped Crosslinker for Multifunctional Shape Memory Polyurethane. Polymers.

[25] Delebecq E., Pascault J.-P., Boutevin B., Ganachaud F. (2013). On the Versatility of Urethane/Urea Bonds: Reversibility, Blocked Isocyanate, and Non-isocyanate Polyurethane. Chem. Rev..

[26] Ireni N.G., Narayan R., Basak P., Raju K.V.S.N. (2016). Poly(thiourethane-urethane-urea) as anticorrosion coatings with impressive optical properties. Polymer.

[27] Li C., Tan J., Li H., Yin D., Gu J., Zhang B., Zhang Q. (2015). Thiol–isocyanate click reaction in a Pickering emulsion: A rapid and efficient route to encapsulation of healing agents. Polym. Chem..

[28] Yan J., Ariyasivam S., Weerasinghe D., He J., Chisholm B., Chen Z., Webster D. (2011). Thiourethane thermoset coatings from bio-based thiols. Polym. Int..

[29] Jia Y., Shi B., Jin J., Li J. (2019). High refractive index polythiourethane networks with high mechanical property via thiol-isocyanate click reaction. Polymer.

[30] Podgórski M., Nair D.P., Chatani S., Berg G., Bowman C.N. (2014). Programmable Mechanically Assisted Geometric Deformations of Glassy Two-Stage Reactive Polymeric Materials. ACS Appl. Mater. Interfaces.

[31] Podgórski M., Wang C., Bowman C.N. (2015). Multiple shape memory polymers based on laminates formed from thiol-click chemistry based polymerizations. Soft Matter.

[32] Nguyen L.-T.T., Truong T.T., Nguyen H.T., Le L., Nguyen V.Q., Van Le T., Luu A.T. (2015). Healable shape memory (thio)urethane thermosets. Polym. Chem..

[33] Gamardella F., Sabatini V., Ramis X., Serra A. (2019). Tailor-made thermosets obtained by sequential dual-curing combining isocyanate-thiol and epoxy-thiol click reactions. Polymer.

[34] Gamardella F., Guerrero F., De la Flor S., Ramis X., Serra A. (2020). A new class of vitrimers based on aliphatic poly(thiourethane) networks with shape memory and permanent shape reconfiguration. Eur. Polym. J..

[35] Gamardella F., Ramis X., De la Flor S., Serra A. (2019). Preparation of poly(thiourethane) thermosets by controlled thiol-isocyanate click reaction using a latent organocatalyst. React. Funct. Polym..

[36] Gamardella F., Muñoz S., De La Flor S., Ramis X., Serra A. (2020). Recyclable Organocatalyzed Poly(Thiourethane) Covalent Adaptable Networks. Polymers.

[37] Konuray O., Areny N., Morancho J.M., Fernàndez-Francos X., Serra À., Ramis X. (2018). Preparation and characterization of dual-curable off-stoichiometric amine-epoxy thermosets with latent reactivity. Polymer.

[38] Konuray O., Liendo F., Fernández-Francos X., Serra A., Sangermano M., Ramis X. (2017). Sequential curing of thiol-acetoacetate-acrylate thermosets by latent Michael addition reactions. Polymer.

[39] Sun X., Gao J.P., Wang Z.Y. (2008). Bicyclic Guanidinium Tetraphenylborate: A Photobase Generator and A Photocatalyst for Living Anionic Ring-Opening Polymerization and Cross-Linking of Polymeric Materials Containing Ester and Hydroxy Groups. J. Am. Chem. Soc..

[40] Gere J.M., Timoshenko S.P. (1985). Mechanics of Materials.

[41] Shonaike G.O., Advani S.G. (2003). Advanced Polymeric Materials: Structure Property Relationships.

[42] Winter H.H., Chambon F. (1986). Analysis of Linear Viscoelasticity of a Crosslinking Polymer at the Gel Point. J. Rheol..

[43] Rogulska M., Kultys A., Olszewska E. (2013). New thermoplastics poly(thiourethane-urethane) elastomers based on hexane-1,6-diyl diisocyanate (HDI). J. Therm. Anal. Calorim..

